# Transient Dysphagia as a Presenting Symptom of Familial Cerebral Cavernous Malformation

**DOI:** 10.7759/cureus.103056

**Published:** 2026-02-05

**Authors:** Madison L Scott, Daniel E Ross

**Affiliations:** 1 Neurology, Alabama College of Osteopathic Medicine, Dothan, USA; 2 Neurology, Emerald Coast Neurology, Pensacola, USA

**Keywords:** autosomal dominant inheritance, cavernous malformation, dysphagia, krit1, susceptibility weighted imaging

## Abstract

Cerebral cavernous malformations (CCMs) are vascular lesions characterized by a collection of thin-walled capillaries with slow blood flow, which are often identified incidentally on MRI. CCMs are the most common cerebral vascular malformation after developmental venous anomalies. Familial CCM (FCCM) is a rare autosomal dominant disorder characterized by several lesions throughout the central nervous system. We report the case of a 47-year-old female patient who presented to the neurology clinic with a chief complaint of transient dysphagia. An MRI of the brain without contrast, including susceptibility-weighted imaging (SWI), demonstrated numerous punctate foci of susceptibility-related signal loss throughout the cerebral and cerebellar hemispheres. Genetic testing revealed a pathogenic KRIT1 mutation, confirming FCCM. The patient’s dysphagia resolved within one month of the initial onset and, fortunately, has not returned. This case highlights an atypical presentation of FCCM and the importance of an extensive workup in patients with unexplained neurologic symptoms.

## Introduction

Cerebral cavernous malformations (CCMs) involve vascular leakage from enlarged capillaries, which can result in neurologic symptoms including seizures, focal neurologic deficits, headaches, and hemorrhagic strokes, to name a few [1,2]. However, 50% to 80% of CCM cases are asymptomatic and are often found incidentally on MRI [1]. CCMs are found in the central nervous system, with supratentorial lesions being more common than infratentorial lesions [2]. The majority of CCM cases involve a single lesion. However, patients with familial CCM (FCCM) typically have several lesions. There are cases of sporadic CCM with multiple lesions, but these are seen concomitantly with developmental venous abnormalities or in patients with radiation-induced CCM [3].

One of the more serious presentations related to CCM is intracerebral hemorrhage. The size and location of the lesion contribute to the likelihood of re-bleeding in the future. Patients with hemorrhage as the initial presentation and those with brainstem CCMs carry the highest risk of subsequent hemorrhage [3]. In FCCM, a prior history of cerebral hemorrhage is associated with an increased risk of rehemorrhage [4]. A study examining the relationship between vitamin D status and hemorrhagic risk in patients with CCMs found an association between low serum 25-hydroxyvitamin D levels and hemorrhagic presentation in CCM [5].

Diagnosis of CCMs can be challenging compared with other vascular diseases, as they are not detectable on cerebral angiography [2]. Lesions that are large enough can be seen on MRI T2 sequences and demonstrate a reticulated lesion similar to a mulberry, with hypointensity surrounding the lesion reflecting the hemosiderin ring [3]. Hemosiderin-sensitive sequences, including gradient echo and susceptibility-weighted imaging (SWI), are the diagnostic tests of choice for identifying cavernous malformations [3]. As seen in the case discussed here, the patient has several CCMs, which were only visualized on SWI sequencing. Cavernous malformations are rare and are often found incidentally, with some of the more common manifestations including headaches, seizures, or symptomatic hemorrhage [6-8]. This case highlights transient dysphagia as an uncommon presentation of FCCM and emphasizes the diagnostic value of SWI and genetic testing in atypical neurologic cases.

## Case presentation

A 47-year-old female patient presented to the neurology clinic with a one-month history of dysphagia. Initially, she only struggled with smaller foods and was not choking, but would have to swallow multiple times to clear her oropharynx. Two weeks prior to her visit, the dysphagia intensified, and she was unable to swallow solid foods. She was able to drink liquids and could tolerate soft foods such as yogurt. She went to the ER when the dysphagia intensified, where CT scans of the head and neck were performed and were unremarkable. Her labs were within normal limits at the ER. She was referred to gastroenterology, which performed an endoscopy and an esophageal dilation without benefit for the dysphagia. She then saw otolaryngology, where a laryngoscopy was performed, which was unremarkable. After these interventions, she was referred to neurology, as the cause for her dysphagia was unclear. Approximately one month after the dysphagia onset, prior to her neurology consultation, the patient’s swallowing was stronger, and she was able to begin eating solids again.

She denied any ptosis, diplopia, ataxia, bowel or bladder incontinence, nausea, or unilateral weakness. She has no history of tobacco or alcohol use. Her past medical history consists of hypothyroidism and psoriasis.

On physical exam, she appeared well nourished, alert, and in no acute distress but mildly anxious. She was oriented to person, place, and time. She was able to stand without difficulty, and her gait was normal. Her neurologic examination, including cranial nerves, strength, sensation, and reflexes, was unremarkable. Based on her clinical presentation before imaging, myasthenia gravis and multiple sclerosis were diagnostic considerations, and further evaluation was pursued.

**Figure 1:**
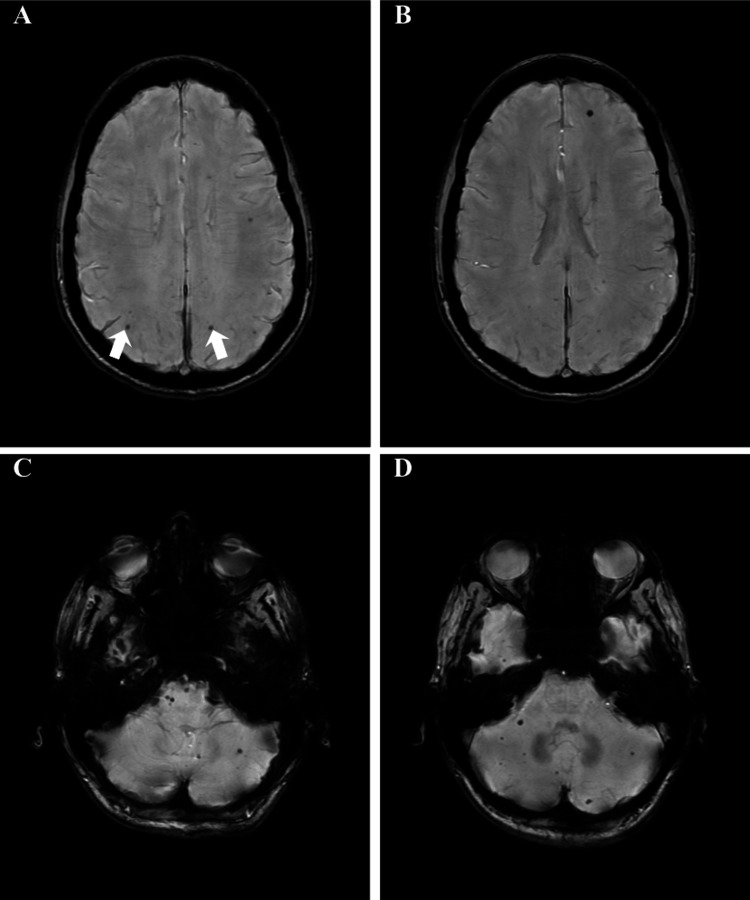
Susceptibility weighted images on MRI of the brain. A: One cavernous malformation is seen in each occipital-parietal lobe, indicated by the white arrows. B: A cavernous malformation is seen in the left frontal lobe. C and D: There are several cavernous malformations seen throughout the brainstem and cerebellum. While the typical appearance of cavernous malformations is mulberry-like clusters, the lesions in this patient are more visually consistent with several microhemorrhages, classifying them as microcavernomas. The radiology report for these MRI slices in this figure included both microhemorrhage and cavernous malformation on the differential diagnoses list. Given this, genetic testing was ordered to assess for familial cerebral cavernous malformation.

Fasting bloodwork revealed that vitamin B6, vitamin E, vitamin B1, vitamin B12, folate, vitamin D, and the myasthenia gravis panel were all within normal limits. Autoimmune testing was unremarkable aside from a low-titer ANA. The MRI of the brain without contrast revealed minimal nonspecific white matter disease, and more notably, it revealed numerous punctate foci of susceptibility effect scattered throughout the cerebral and cerebellar hemispheres, best seen on SWI (Figure 1). After the MRI, the differential included microhemorrhage, microcalcification, numerous cavernous malformations, and/or neurocysticercosis. A lesion was observed in the patient’s brainstem and was felt to be associated with her transient dysphagia.

**Figure 2:**
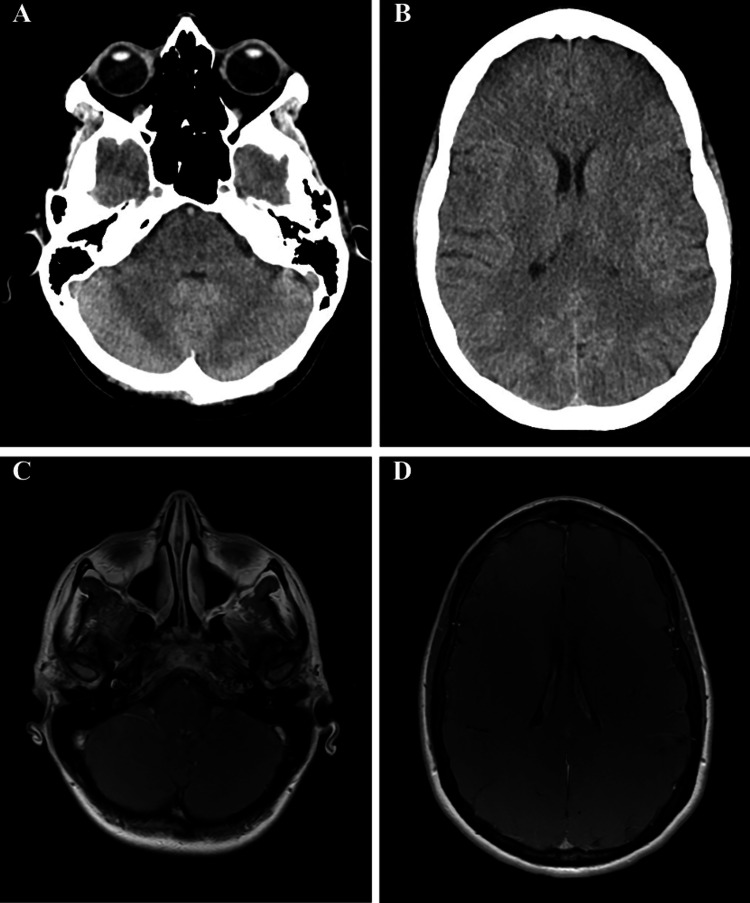
CT and MRI of the brain with contrast. Panels A and B show a non-contrast CT assessing for calcifications, while panels C and D show a contrast-enhanced MRI evaluating for abnormal enhancement. No calcifications or abnormal enhancement were observed.

Given these findings, a CT of the brain was ordered to assess for calcifications, an MRI of the brain with contrast was ordered to assess for enhancement, and genetic testing was ordered, including a CCM panel. Her CT of the head did not demonstrate calcifications, and there was no abnormal enhancement on MRI (Figure 2), making neurocysticercosis unlikely.

Her genetic testing revealed a pathologic variant of the KRIT1 gene, which is associated with autosomal dominant CCM. The CCM2 and PDCD10 genes were also assessed, but were not pathogenic.

Given that spinal cord lesions can also be seen with FCCM, MRIs of the cervical and thoracic spine were ordered, and fortunately, they did not reveal evidence for CCMs in the spinal cord.

As for clinical management, we discussed the likelihood of her children developing this disorder, as KRIT1 has an autosomal dominant inheritance pattern. The patient had no known family history of CCM before her diagnosis, but she likely inherited the mutated gene from one of her parents. She was advised not to participate in activities with a high likelihood of head trauma, given her increased risk of intracerebral hemorrhage that is associated with FCCM. It was also recommended that she continue eating a healthy diet with foods rich in vitamin D, as emerging literature suggests an association between low vitamin D levels and hemorrhaging in CCM cases [5]. She was referred to rheumatology for the positive ANA.

## Discussion

The prevalence of CCMs has been reported to be 0.1% to 0.8% of the population [2]. The mean age for CCMs is around 37 years old, but they can present at any age [2]. FCCMs are inherited in an autosomal dominant manner and account for approximately 20% of all cavernous malformation cases [1]. FCCM is linked with heterozygous loss-of-function mutations in KRIT1, CCM2, or PDCD10 genes [9]. KRIT1 mutations disrupt endothelial integrity and angiogenesis, contributing to lesion formation [2]. Individuals with a mutation in the CCM2 gene are more likely to be asymptomatic and have fewer lesions than those with mutations in KRIT1 or PDCD10 [9]. CCM2 normally functions by regulating heart and vessel formation and integrity, along with stabilizing endothelial cell junctions [9]. The PDCD10 gene has multiple functions, including regulating apoptotic pathways, is required for angiogenesis, and is involved in KDR/VEGFR2 activity, to name a few [2]. With KRIT1 mutations, a single copy of the inherited mutation alone is not enough to cause the disease. It is suggested that a “second hit” or a sporadic mutation in addition to the inherited mutation is necessary for lesion development [3]. As an autosomal dominant heterozygous mutation causes FCCM, those affected have a 50% chance of passing the disorder on to offspring. While mutations in KRIT1, CCM2, or PDCD10 make up the majority of FCCM cases, 5% to 15% of cases cannot be explained by these genes [10].

This case is notable because it illustrates transient dysphagia as the presenting clinical manifestation of FCCM, resolving without intervention, in contrast to prior reports that describe persistent dysphagia associated with brainstem cavernous malformations that required surgical intervention [11-14]. Given the complexity of the brainstem, lesions here have the potential to cause more severe symptoms. In this case, the lesion was small and, to our knowledge, had not hemorrhaged previously. In prior reports, surgical intervention took place in patients with much larger brainstem cavernomas that caused dysphagia [11-13, 15]. A prior report described a large brainstem cavernous malformation causing severe and persistent swallowing dysfunction, including recurrent aspiration pneumonia secondary to pharyngeal phase impairment [14]. In contrast, the present case demonstrates that smaller cavernous malformations can still have significant clinical implications but are capable of symptom resolution, likely through resorption of the hemorrhage and decreased localized inflammation. The brainstem, specifically the medulla oblongata, contains the ambiguus nucleus, which controls cranial nerves IX and X, responsible for producing and coordinating the swallowing sequence [16, 17]. Due to the anatomical convergence of both afferent and efferent pathways for swallowing in the brainstem, even small lesions can have large clinical impacts [16]. This case is unique, given that the dysphagia gradually improved and resolved independent of medical intervention, likely because of the size of the lesion.

Swallowing is a complex process that requires the activation and coordination of over 25 pairs of muscles, intact pharyngeal sensation, and central neural control originating from the brainstem, cerebellum, basal ganglia, and cerebral cortex [16]. With this being said, dysphagia can be caused by several etiologies. Neurogenic dysphagia occurs when neurologic damage disrupts any component of the swallowing pathway [16]. Neurogenic dysphagia can be seen in several diseases, including stroke, amyotrophic lateral sclerosis, Parkinson’s disease, dementia, neuromuscular disorders, and lesions of the brainstem [16]. While neurogenic dysphagia is commonly associated with vascular, neurodegenerative, and demyelinating disorders, brainstem cavernous malformations represent a rarely quantified cause.

Three treatment options for cavernous malformations include microsurgical resection, stereotactic radiosurgery, and conservative management [18]. Resecting brainstem cavernous malformation lesions poses a greater risk than resection of other cerebral lesions [18]. A prior study suggested that hemorrhages associated with cerebral malformations can resolve over time as the blood products are resorbed, with corresponding attenuation of symptoms [18]. The risk of rehemorrhage is higher in cases of brainstem lesions than in other cerebral lesions [19]. A longitudinal study revealed that the risk of recurrent intracranial hemorrhage or focal neurologic deficit from a cavernous malformation is higher than the risk of an initial event, higher in women than men, and declines over five years [20]. Unlike previously reported brainstem lesions that required surgical intervention, this lesion stabilized spontaneously, allowing symptom resolution.

Within the setting of existing literature on brainstem cavernous malformations, neurogenic dysphagia, and the natural history of FCCM, this case expands the clinical spectrum to include transient, self-resolving dysphagia.

## Conclusions

FCCMs can remain asymptomatic but may cause neurologic symptoms depending on the size and location of the lesion. In this case, the patient’s dysphagia resolved spontaneously, and she has remained clinically stable without symptom recurrence. This case report highlights the importance of a comprehensive neurologic evaluation and genetic testing in patients with atypical presentations and no previously identified family history of neurologic deficits. Additionally, it contributes to the existing literature on neurogenic dysphagia associated with cavernous malformation by documenting symptom resolution independent of medical or surgical intervention.

## References

[1] Choquet H, Pawlikowska L, Lawton MT, Kim H (2015). Genetics of cerebral cavernous malformations: current status and future prospects. J Neurosurg Sci.

[2] Zafar A, Quadri SA, Farooqui M (2019). Familial cerebral cavernous malformations. Stroke.

[3] Flemming KD, Lanzino G (2020). Cerebral cavernous malformation: what a practicing clinician should know. Mayo Clin Proc.

[4] Weinsheimer S, Nelson J, Abla AA (2023). Intracranial hemorrhage rate and lesion burden in patients with familial cerebral cavernous malformation. J Am Heart Assoc.

[5] Flemming KD, Kumar S, Brown RD Jr, Singh RJ, Whitehead K, McCreath L, Lanzino G (2020). Cavernous malformation hemorrhagic presentation at diagnosis associated with low 25-hydroxy-vitamin D level. Cerebrovasc Dis.

[6] Caton MT, Karsonovich T, Shenoy VS (2025). Cerebral Cavernous Malformations. https://pubmed.ncbi.nlm.nih.gov/30844171/.

[7] D'Souza D, BenSalem A, Feger J (2026). Cerebral cavernous venous malformation. https://doi.org/10.53347/rID-1064.

[8] Dalyai RT, Ghobrial G, Awad I (2011). Management of incidental cavernous malformations: a review. Neurosurg Focus.

[9] Snellings DA, Hong CC, Ren AA (2021). Cerebral cavernous malformation: from mechanism to therapy. Circ Res.

[10] Kim J (2016). Introduction to cerebral cavernous malformation: a brief review. BMB Rep.

[11] Candanedo C, Moscovici S, Spektor S (2019). Medulla oblongata cavernoma removal through a lazy far lateral approach: operative video and technical nuances. Neurosurg Focus Video.

[12] Matsushima K, Kohno M, Bertalanffy H (2019). Microsurgical resection of medullary cavernoma via the olivary zone by the retrosigmoid supracondylar approach. Neurosurg Focus Video.

[13] Zhang S, Lin S, Hui X, Li H, You C (2017). Surgical treatment of cavernous malformations involving medulla oblongata. J Clin Neurosci.

[14] Yoshimatsu Y, Tobino K, Kawabata T (2021). Hemorrhaging from an intramedullary cavernous malformation diagnosed due to recurrent pneumonia and diffuse aspiration bronchiolitis. Intern Med.

[15] London D, Lieberman S, Tanweer O, Pacione D (2020). Transclival approach for resection of a pontine cavernous malformation: 2-dimensional operative video. Oper Neurosurg.

[16] D'Alatri L, Marchese MR, Tizio A, Galli J (2025). Pathophysiology and etiology of brainstem-related dysphagia. Audiol Res.

[17] Jean A (2001). Brain stem control of swallowing: neuronal network and cellular mechanisms. Physiol Rev.

[18] Mouchtouris N, Chalouhi N, Chitale A, Starke RM, Tjoumakaris SI, Rosenwasser RH, Jabbour PM (2015). Management of cerebral cavernous malformations: from diagnosis to treatment. ScientificWorldJournal.

[19] Al-Shahi Salman R, Hall JM, Horne MA (2012). Untreated clinical course of cerebral cavernous malformations: a prospective, population-based cohort study. Lancet Neurol.

[20] Taslimi S, Modabbernia A, Amin-Hanjani S, Barker FG 2nd, Macdonald RL (2016). Natural history of cavernous malformation: systematic review and meta-analysis of 25 studies. Neurology.

